# Primary care randomised controlled trial of a tailored interactive website for the self-management of respiratory infections (Internet Doctor)

**DOI:** 10.1136/bmjopen-2015-009769

**Published:** 2016-04-20

**Authors:** Paul Little, Beth Stuart, Panayiota Andreou, Lisa McDermott, Judith Joseph, Mark Mullee, Mike Moore, Sue Broomfield, Tammy Thomas, Lucy Yardley

**Affiliations:** 1 Primary Care Group, Primary Care and Population Sciences Unit, University of Southampton, Southampton, UK; 2 Centre for the Applications of Health Psychology, University of Southampton, Southampton, UK; 3 Research Design Service South Central, Primary Care and Population Sciences Unit, University of Southampton, Southampton, UK

## Abstract

**Objective:**

To assess an internet-delivered intervention providing advice to manage respiratory tract infections (RTIs).

**Design:**

Open pragmatic parallel group randomised controlled trial.

**Setting:**

Primary care in UK.

**Participants:**

Adults (aged ≥18) registered with general practitioners, recruited by postal invitation.

**Intervention:**

Patients were randomised with computer-generated random numbers to access the intervention website (intervention) or not (control). The intervention tailored advice about the diagnosis, natural history, symptom management (particularly paracetamol/ibuprofen use) and when to seek further help.

**Outcomes:**

Primary: National Health Service (NHS) contacts for those reporting RTIs from monthly online questionnaires for 20 weeks. Secondary: hospitalisations; symptom duration/severity.

**Results:**

3044 participants were recruited. 852 in the intervention group and 920 in the control group reported 1 or more RTIs, among whom there was a modest increase in NHS direct contacts in the intervention group (intervention 37/1574 (2.4%) versus control 20/1661 (1.2%); multivariate risk ratio (RR) 2.25 (95% CI 1.00 to 5.07, p=0.048)). Conversely, reduced contact with doctors occurred (239/1574 (15.2%) vs 304/1664 (18.3%); RR 0.71, 0.52 to 0.98, p=0.037). Reduction in contacts occurred despite slightly longer illness duration (11.3 days vs 10.7 days, respectively; multivariate estimate 0.60 days longer (−0.15 to 1.36, p=0.118) and more days of illness rated moderately bad or worse illness (0.52 days; 0.06 to 0.97, p=0.026). The estimate of slower symptom resolution in the intervention group was attenuated when controlling for whether individuals had used web pages which advocated ibuprofen use (length of illness 0.22 days, −0.51 to 0.95, p=0.551; moderately bad or worse symptoms 0.36 days, −0.08 to 0.80, p=0.105). There was no evidence of increased hospitalisations (risk ratio 0.25; 0.05 to 1.12; p=0.069).

**Conclusions:**

An internet-delivered intervention for the self-management of RTIs modifies help-seeking behaviour, and does not result in more hospital admissions due to delayed help seeking. Advising the use of ibuprofen may not be helpful.

**Trial registration number:**

ISRCTN91518452.

**Strengths and limitations of this study**

- This is, to the best of our knowledge, the only substantial trial to date to address the effectiveness of support for the management of respiratory infections using the internet.
- The rate of uptake following invitation was low, but is what would be expected for a free-standing internet-delivered intervention and 70% follow-up was achieved, which is high for a free-standing internet intervention—and there was little evidence of attrition bias.
- The primary outcome had to be changed to monthly questionnaires since the intervention development had to take account of the context of the provision of National Health Service (NHS) Direct, and the monthly self-report data was the only source of data about NHS Direct contacts (in addition to documenting episodes that clinicians did not include in the records), but recall of contacts made during an infection experienced in the previous month are likely to suffer minimal recall bias.
- Participants were less deprived than non-participants, but controlling for deprivation made little difference to the estimates and there was no significant interaction of the intervention with deprivation.
- The number of participants who experienced one or more respiratory tract infections was lower than expected, which will have reduced the power to detect differences.

## Background

Most people suffer a respiratory tract infection (RTI) every year, many suffering more than once, with 20–30% of the population consulting primary care at least once each year, which represents a significant call on healthcare resources.^1^ ^2^ However, in most cases, RTIs do not present a serious threat to the patient's health and with access to the right information many illnesses could be self-managed at home. This is particularly important as, unfortunately, when a doctor is consulted, antibiotics are normally given.^1^ Provision of such information prior to consultations could potentially result in patients having improved symptom control, lower attendance at general practitioner (GP) surgeries and reduced antibiotic prescriptions—which could be one important tool in the fight against antibiotic resistance.^3^ A systematic review has documented several trials that have used information to modify consultations for RTIs among children.^4^ However, there were only three older trials (the last published in 1991) that addressed the issue of providing specific information prior to consultation for RTIs.^4^ Studies in adults also demonstrate that providing information booklets may help modify consultation behaviour,^5–7^ but a wide range of symptoms and conditions were assessed in the latter studies, so the precise role in interventions for modifying consultations for RTIs is less clear.

Booklets are no longer likely to be distributed as a source of advice regarding the self-management of respiratory infection given the widespread and growing access to the internet as a source of information prior to consulting—with more than 80% of families currently having access to the internet (rising by 5% each year). Web-based interventions can enable patients to access reliable self-care information from their home, make an informed decision on how best to manage their symptoms and decide whether they need to visit their doctor. Recently, a trial has reported that advice to use ibuprofen resulted in both poor symptom control (more prolonged illness) and increased complication—presumably by interfering with the inflammatory and immune response.^8^ A potential problem about providing self-management advice is that patients might be encouraged to self-manage serious infections inappropriately (ie, when they really need to see the doctor), and so develop complications unnecessarily. This is a major concern for doctors and patients^9–11^—highlighting the importance of good safety-netting advice (ie, advice about when to consult further) and the need to document the impact of interventions on hospital admissions. However, it is also plausible that good self-management advice about appropriate early assessment of more severe illness could reduce hospital admissions.

We have developed a theoretically informed internet-delivered intervention to manage RTIs among adults (‘The Internet Doctor’) that we have shown in a small exploratory trial results in higher levels of satisfaction, enablement and understanding of illness.^12^ We report a larger trial of this website to address whether consultation behaviour can be modified, and to document potential harms (including hospital admissions) over a 1-year period.

## Methods

We used procedures very similar to our previous leaflet trial.^6^ A random selection of adults in the computerised practice registers from 35 practices in southern England were identified by the practice staff and letters sent to patients inviting them to participate. Patients willing to participate were asked to log on to the website to confirm consent. Patients were also given contact details to enable them to email or talk to the research team before agreeing to participate, or if they had problems logging in. Only one participant per household could participate.

### Changes to the protocol

We originally specified a 12-month period for measuring the primary outcome, but in developing the intervention, we needed to incorporate not just advice to see the GP but also advice to use National Health Service (NHS) Direct, and therefore, to document NHS Direct contacts. We had not anticipated this and so required self-report of the monthly data as our primary outcome. To provide monthly follow-ups for a year would then have had two effects—engagement of participants would have been much more difficult and much more resource intensive than originally anticipated. The most meaningful and feasible assessment of the primary outcome was, therefore, the monthly reports of consulting their GP for those individuals who reported a respiratory infection (the intervention was not designed to help those who did not suffer an infection).

*Inclusion criteria*. Adult patients (aged 18+ years) from GPs computerised lists.

*Exclusion criteria.* Patients with severe mental problems (eg, major uncontrolled depression/schizophrenia; dementia; severe mental impairment—unable to complete outcomes) or terminally ill.

*Randomisation*. Once logged in, patients were randomised automatically by the website using computer generated random numbers to one of the following groups:

- Access to an interactive website providing tailored advice; this was reinforced by email prompts and reminders to use the website; patients were given information about the natural history, self-care advice, and advice about the use of over-the-counter medication. Outcome measures were documented online by participants following email prompts each month.
- Normal care (as the control group, outcome measures were collected online, but access to the tailored advice website was at the end of the trial).

Randomisation was not stratified, with no blocking, and participants were blind to their randomisation group at the point of consent (but clearly could not be blinded once they knew their randomisation group).

### Study groups

#### Intervention group

Participants had access to the internet-delivered intervention for 20 weeks. On logging onto the website, users could select tailored advice on (1) whether and why they need/do not need to consult the GP and (2) how to self-care for RTIs. Patients selecting consultation advice completed questions about their symptoms and medical history, and were then presented with tailored advice recommending either self-management (for mild symptoms), for more severe symptoms (eg, haemoptysis, prolonged fever) phoning the ‘NHS Direct’ helpline, which provided nurse-led advice about the need to seek further medical help, or alternatively, seeking medical attention immediately (for symptoms potentially posing serious risks, eg, reduced consciousness level, chest pain). Patients were given the opportunity to challenge this advice by selecting further in-depth information about the symptoms of common complications or serious illness compatible with their symptoms, and by clicking on frequently asked questions (eg, regarding the need for antibiotics and typical time-course of symptoms). The self-care section provided options to select advice on self-management without medication (including rest, fluid intake) or with medication. For those who wanted to take medication, over-the-counter remedies were recommended as an effective and preferable alternative to seeking antibiotics from the GP, and in particular, optimising the use of paracetamol and encouraging the use of ibuprofen. The website was theory-based, addressing all components of the common-sense model of self-regulation of illness^13^ (ie, perceived symptoms, cause, timeline, physical and emotional consequences and the possibility for control/cure), and used the principles of social cognitive theory^14^ to address expected outcomes of consultation and self-care, and build self-confidence for self-care. Extensive qualitative piloting^15^ established that the website was accessible to people with very limited education and no previous computer experience, and quantitative piloting in several hundred people indicated that it increased confidence when self-managing a RTI, and had the potential to reduce consultations.^12^

#### Control group (normal care)

As in the intervention group, the control group had access to the GP/practice in the normal way for respiratory illnesses and influenza-like-illness (ILI). The control group was offered access to the website at the end of the study to minimise resentful demoralisation.^16^

### Primary outcome

#### GP consultations

We hypothesised that the intervention would reduce the number of contacts with GPs for individuals who suffered a RTI. Patients were prompted by email to log onto the website monthly, every 4 weeks, until 20 weeks (ie, weeks 4, 8, 16, 20) to complete questionnaires about illnesses during the last month—since the duration of symptoms can be remembered reliably over a period of a few weeks.^17^ ^18^

We also performed an assessment of the consultations that were recorded in primary care by a blinded assessment of the primary care records. Although this does not capture all contacts with health professionals (and also does not capture contacts with NHS Direct) it has been shown to be reliable.^19^

### Secondary outcomes

The use of antibiotics was documented as prescription of antibiotics, from patient records.

For each episode, the index person also documented: whether they contacted NHS Direct for phone-based advice; the nature of the infection; the duration of symptoms rated moderately bad (which we have shown in previous research is a useful outcome and sensitive to change for individuals,^18^ and can be remembered reliably over a period of a few weeks^17^ ^18^); the number of days where work/normal activities were impaired;^18^ and smoking status.

Patients were also asked to complete measures of their symptoms and concern about them at the time of illness, levels of health anxiety, consulting preferences, and attitudes to the intervention; a full analysis of these potential mediators and moderators of outcomes will be presented in a process analysis in a future paper.

#### Sociodemographic and comorbidity data

We collected age, gender and educational level from the participant online and prior comorbidities and consultations from the notes review.

#### Sample size calculation

We estimated that a trial among a minimum of 2266 patients would allow us to detect a 25% reduction in attendance with RTIs (20% vs 15% requires 906 per group, with completed outcomes or 2266 allowing for 20% loss to follow-up; for α=0.05 and β=0.2), and a 0.2 standardised effect size for continuous outcomes.

#### Analysis

We performed an intention-to-treat analysis, and the syntax was written blind as to group. No interim analysis was performed. The proportions attending with RTI in the intervention and normal care groups were evaluated using logistic regression to calculate ORs (which were converted to risk ratios using the formula of Zhang^20^), with CIs. Outcomes measured on a continuous scale (duration and severity of symptoms) were analysed using multiple linear regression. All continuous outcome variables were checked for the assumption of normality of residuals. The models controlled for variables likely to predict consultation: gender, age, highest educational qualification, smoking status, whether there were children aged under 16 years living in the household, any comorbid condition, the number of times the patient reported consulting a doctor about an RTI in the 12 months prior to the study, and index of multiple deprivation (IMD uses post codes to estimate deprivation across a number of domains; https://www.gov.uk/government/statistics/english-indices-of-deprivation-2010). Given previous findings of increased symptom burden when health professionals give advice to use ibuprofen,^8^ and the findings of increased symptom burden in the intervention group of the current study, a post hoc secondary analysis explored the impact of controlling for whether pages advocating the use of ibuprofen had been viewed.

## Results

**Figure 1:**
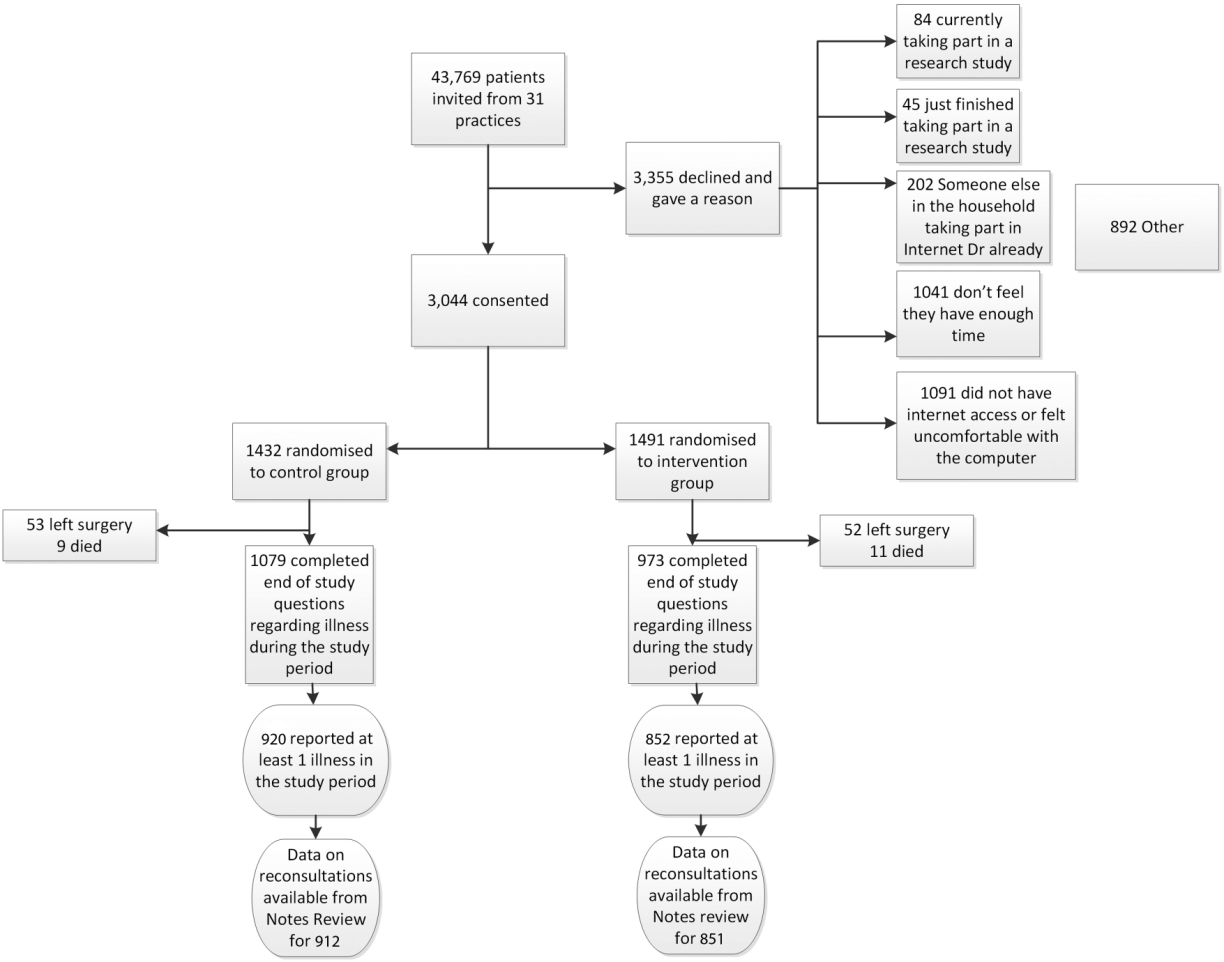
CONSORT diagram.

Totally, 43 769 patients were invited, of whom 3044 participants consented (from 17 January 2012 to 20 October 2013), and 3355 gave reasons for declining (commonly not enough time, or insufficient access to the internet, or uncomfortable using computers, but also a variety of other reasons; see figure 1). Table 1 demonstrates that the groups were well balanced for a range of variables (and table 2 shows this for those who reported at least one respiratory infection during follow-up). Although groups in the study were well balanced for deprivation, those who agreed to take part were less deprived than non-participants (IMD score 16.1 (SD 11.1), hence results controlled for IMD score.

**Table 1:** Baseline characteristics*

	Control	Intervention
Female	779/1432 (54.4%)	816/1490 (54.8%)
Age	57.14 (13.1)	56.78 (13.5)
Ever smoked	699/1425 (49.1%)	688/1483 (46.4%)
IMD score	12.6 (7.9)	12.9 (8.1)
Comorbid condition	511/1418 (36.0%)	549/1481 (37.1%)
Number of times consulted a doctor about RTI in the previous year	0.50 (1.2)	0.54 (1.2)
Household composition (%)		
Alone	178/1432 (12.4)	191/1489 (12.8)
Spouse/partner	963/1432 (67.3)	1015/1489 (68.2)
Other adult(s)	147/1432 (10.3)	145/1489 (9.7)
Children aged under 16 years	144/1432 (10.1)	138/1489 (9.3)
Highest qualifications		
No formal educational qualifications	108/1432 (7.5)	121/1490 (8.1)
Cses/o'levels/gcses (or similar)	265/1432 (18.5)	279/1490 (18.7)
A'levels (or similar)	151/1432 (10.5)	157/1490 (10.5)
Diploma/other vocation qualification	317/1432 (22.1)	322/1490 (21.6)
Degree	218/1432 (15.2)	244/1490 (16.4)
Postgraduate or professional qualification	373/1432 (26.1)	367/1490 (24.6)

*Data are means (SD) or numbers (%).

IMD, index of multiple deprivation; RTI, respiratory tract infection.

**Table 2:** Baseline characteristics of participants who reported at least one respiratory tract infection (RTI)*

	Control	Intervention
Female	506/920 (55.0%)	491/852 (57.6%)
Age	56.28 (12.95)	56.76 (12.93)
Ever smoked	448/918 (48.8%)	393/850 (46.2%)
Comorbid condition	329/912 (36.1%)	324/850 (38.1%)
Number of times consulted a doctor about RTI in the previous year	0.54 (1.17)	0.54 (1.19)
Household composition (%)		
Alone	110/920 (12.0)	98/851 (11.5)
Spouse/partner	612/920 (66.5)	589/851 (69.2)
Other adult(s)	96/920 (10.4)	87/851 (10.2)
Children aged under 16 years	102/920 (11.1)	77/851 (9.1)
Highest qualifications		
No formal educational qualifications	66/920 (7.2)	62/852 (7.3)
Cses/o'levels/gcses (or similar)	166/920 (18.0)	143/852 (16.8)
A'levels (or similar)	102/920 (11.1)	88/852 (10.3)
Diploma/other vocation qualification	203/920 (22.1)	196/852 (23.0)
Degree	139/920 (15.1)	148/852 (17.4)
Postgraduate or professional qualification	244/920 (26.5)	215/852 (25.2)

*Data are means (SD) or numbers (%).

Table 3 documents a modest increase in contacts for NHS Direct among those who had an RTI in the intervention group (37/1574 (2.4%) versus 20/1661 (1.2%), multivariate risk ratio (RR) 2.25 (1.00 to 5.07, p=0.048), but reduced contact with doctors (239/1574 (15.2%) vs 304/1664 (18.3%), risk ratio 0.71, 95% CI 0.52 to 0.98, p=0.037).

**Table 3:** Monthly reports of health service use and duration of illness (weeks 4, 8, 12, 16 and 20) for participants who reported at least one respiratory infection during the 20 weeks

	Control	Intervention	Univariate risk ratio (95% CI; p value)	Multivariate risk ratio* (95% CI; p=value)
Reported episodes of respiratory tract infection	1665/5697 (29.23%)	1578/5291 (29.82%)	1.03 (0.93 to 1.12; p=0.566)	1.04 (0.94 to 1.14; p=0.461)
Of those who reported a respiratory tract infection				
Saw a doctor about illness (as a proportion of the number of episodes)	304/1664 (18.27%)	239/1574 (15.18%)	0.75 (0.56 to 1.01; p=0.061)	0.71 (0.52 to 0.98; p=0.037)
Contacted NHS Direct about illness	20/1661 (1.20%)	37/1574 (2.35%)	2.34 (1.07 to 5.10; p=0.034)	2.25 (1.00 to 5.07; p=0.048)
			Difference (95% CI; p value)	
Length of illness (days)	10.68 (9.45)	11.30 (9.89)	0.58 (−0.15 to 1.30; p=0.119)	0.60 (−0.15 to 1.36; p=0.118)
Days moderately bad or worse NHS, National Health Service	4.00 (5.48)	4.59 (6.88)	0.47 (0.03 to 0.92; p=0.035)	0.52 (0.06 to 0.97; p=0.026)

*Multivariate model controls for gender, age, highest educational qualification, smoking status, whether there are children aged under 16 years living in the household, any comorbid condition, index of multiple deprivation score, and the number of times the patient reported consulting a doctor about an RTI in the 12 months prior to the study.

### 

#### Possible harms

The reduction in contacts with doctors occurred despite slightly longer duration of illness (>11.3 vs 10.7 days); multivariate estimate 0.60 days longer (−0.15 to 1.36, p=0.118) and more days experienced of moderately bad or worse illness 4.59 vs 4.00 days (multivariate estimate 0.52 days; 0.06 to 0.97, p=0.026). The latter estimates of increased symptom burden were reduced when controlling for whether individuals used ibuprofen from the pages on the website (length of illness 0.22, −0.51 to 0.95, p=0.551; moderately bad or worse symptoms 0.36, −0.08 to 0.80, p=0.105). There was no evidence that self-management advice resulted in delayed consultations for serious illnesses (eg, lobar pneumonia; meningitis; septicaemia), and hence, increased hospitalisations: in fact there were reduced hospitalisations, albeit not statistically significant, both in the shorter term (20 weeks) and longer term (1 year) (tables 4–7).

**Table 4:** Health service use recorded in primary care records in the 20 weeks following the date of consent for participants who reported at least one episode of respiratory tract infection (RTI)

	Control (%)	Intervention (%)	Univariate risk ratio (95% CI; p=value)	Multivariate risk ratio* (95% CI; p=value)
Any consultations	98/912 (10.8)	88/851 (10.3)	0.96 (0.73 to 1.26; p=0.782)	0.89 (0.65 to 1.23; p=0.514)
Any antibiotic prescriptions	66/880 (7.5)	64/827 (7.7)	1.03 (0.74 to 1.43; p=0.853)	0.94 (0.64 to 1.38; p=0.759)
Any hospitalisations	7/823 (0.9)	1/765 (0.1)	0.15 (0.02 to 1.24; p=0.079)	0.13 (0.02 to 1.11; p=0.062)
Any referrals	10/824 (1.2)	8/771 (1.0)	0.86 (0.34 to 2.14; p=0.740)	0.77 (0.26 to 2.24; p=0.625)

*Multivariate model controls for gender, age, highest educational qualification, smoking status, whether there are children aged under 16 years living in the household, any comorbid condition, index of multiple deprivation score, and the number of times the patient reported consulting a doctor about an RTI in the 12 months prior to the study.

**Table 5:** Health service use recorded in primary care records in the 12 months following the date of consent for patients who experience at least one episode of respiratory tract infection (RTI) in the first 20 weeks

	Control	Intervention	Univariate risk ratio (95% CI; p=value)	Multivariate risk ratio (95% CI; p=value)
Any reconsultations	176/912 (19.3%)	164/851 (19.3%)	0.99 (0.82 to 1.16; p=0.989)	0.93 (0.73 to 1.16; p=0.509)
Number of reconsultations†	0.36 (1.01)	0.33 (0.85)	0.91 (0.71, 1.17; p=0.475)	0.94 (0.72, 1.21; p=0.619)
Any antibiotic prescriptions	115/851 (13.5%)	107/794 (13.5%)	0.99 (0.78 to 1.30; p=0.982)	1.00 (0.74 to 1.33; p=0.997)
Any hospitalisations	8/748 (1.1%)	1/689 (0.2%)	0.14 (0.02 to 1.08; p=0.059)	0.13 (0.02 to 1.05; p=0.056)
Any referrals	14/750 (1.9%)	12/699 (1.7%)	0.92 (0.43 to 1.96; p=0.830)	0.87 (0.35 to 2.16; p=0.799)

*Multivariate model controls for gender, age, highest educational qualification, smoking status, whether there are children aged under 16 years living in the household, any comorbid condition, index of multiple deprivation score, and the number of times the patient reported consulting a doctor about an RTI in the 12 months prior to the study.

†Reported as the mean (SD). The median is 0 and the IQR is (0, 0). The range is 0–8.

**Table 6:** Health service use in the 20 weeks following the date of consent based on review of primary care notes

	Control	Intervention	Univariate risk ratio (95% CI; p=value)	Multivariate risk ratio (95% CI; p=value)
Any reconsultations	126/1418 (8.89%)	118/1483 (7.96%)	0.90 (0.71 to 1.14; p=0.368)	0.95 (0.79 to 1.15; p=0.612)
Number of reconsultations†	0.18 (0.75)	0.16 (0.66)	0.88 (0.64 to 1.21; p=0.434)	0.97 (0.69 to 1.35; p=0.854)
Any antibiotic prescriptions	86/1378 (6.24%)	83/1448 (5.73%)	0.92 (0.68 to 1.23; p=0.569)	0.88 (0.63 to 1.24; p=0.473)
Any hospitalisations	8/1301 (0.61%)	2/1368 (0.15%)	0.25 (0.05 to 1.12; p=0.069)	0.24 (0.05 to 1.13; p=0.072)
Any referrals	10/1302 (0.77%)	10/1375 (0.73%)	0.95 (0.39 to 2.26; p=0.903)	0.98 (0.37 to 2.59; p=0.965)

*Multivariate model controls for gender, age, highest educational qualification, smoking status, whether there are children aged under 16 years living in the household, any comorbid condition, index of multiple deprivation score and the number of times the patient reported consulting a doctor about an respiratory tract infection in the 12 months prior to the study.

†Reported as the mean (SD). The median is 0 and the IQR is (0, 0). The range is 0–8.

**Table 7:** Health service use in the 12 months following the date of consent based on review of primary care notes

	Control	Intervention	Univariate risk ratio (95% CI; p=value)	Multivariate risk ratio (95% CI; p=value)
Any reconsultations	242/1418 (17.07%)	249/1483 (16.79%)	0.98 (0.83 to 1.15; p=0.843)	0.85 (0.65 to 1.12; p=0.259)
Number of reconsultations†	0.30 (0.88)	0.28 (0.77)	0.92 (0.75 to 1.14; p=0.456)	0.97 (0.79 to 1.21; p=0.806)
Any antibiotic prescriptions	156/1332 (11.71%)	155/1389 (11.16%)	0.95 (0.77 to 1.17; p=0.651)	0.97 (0.76 to 1.23; p=0.811)
Any hospitalisations	11/1189 (0.92%)	4/1239 (0.32%)	0.35 (0.11 to 1.09; p=0.071)	0.35 (0.11 to 1.10; p=0.073)
Any referrals	16/1192 (1.34%)	15/1249 (1.20%)	0.89 (0.44 to 1.80; p=0.755)	1.11 (0.48 to 2.52; p=0.808)

*Multivariate model controls for gender, age, highest educational qualification, smoking status, whether there are children under 16 living in the household, any comorbid condition, index of multiple deprivation score, and the number of times the patient reported consulting a doctor about a respiratory tract infection in the 12 months prior to the study.

†Reported as the mean (SD). The median is 0 and the IQR is (0, 0). The range is 0–8.

Analysis of the follow-up data from the notes review for the whole sample is shown in tables 6 and 7; as expected, since most such individuals did not have a respiratory infection, there was no clear evidence of a reduction in consultations. The characteristic of those followed-up and not followed-up, were also similar (table 8).

**Table 8:** Characteristics of participants followed up and not followed up

	Did not complete 20-week follow up questions	Did complete 20-week follow up questions
Control group	353/871 (40.5%)	1079/2052 (52.6%)
Intervention group	518/871 (59.5%)	973/2052 (47.4%)
Female	489/871 (56.1%)	1106/2051 (53.9%)
Age	52.9 (14.9)	58.7 (12.1)
Ever smoked	428/862 (49.7%)	959/2046 (46.9%)
IMD score	13.8 (9.0)	12.3 (7.4)
Comorbid condition	290/ 861 (33.7%)	770/2038 (37.8%)
Number of times consulted a doctor about RTI in the previous year	0.71 (1.4)	0.4 (1.1)
Household composition		
Alone	113/871 (13.0%)	256/2050 (12.5%)
Spouse/partner	550/871 (63.2%)	1428/2050 (70.0%)
Other adult(s)	101/871 (11.6%)	191/2050 (9.3%)
Children aged under 16 years	107/871 (12.3%)	175/2050 (8.5%)
Highest qualifications		
No formal educational qualifications	76/871 (8.7%)	153/2051 (7.5%)
Cses/o'levels/gcses (or similar)	180/871 (20.7%)	364/2051 (17.8%)
A'levels (or similar)	93/871 (10.7%)	215/2051 (10.5%)
Diploma/other vocation qualification	191/871 (21.9%)	448/2051 (21.8%)
Degree	142/871 (16.3%)	320/2051 (15.6%)
Postgraduate or professional qualification	189/871 (21.7%)	551/2051 (26.9%)

IMD, index of multiple deprivation; RTI, respiratory tract infection.

## Discussion

This is, to our knowledge, the only substantial trial to date to address the effectiveness of support for the management of respiratory infections using the internet. Although relatively limited follow-up was possible (20 weeks) there was reduced contact with GPs, and possibly a longer term reduction in hospital admissions. There was a slight increase in contact with NHS Direct, consistent with the advice given by the intervention for management of more severe symptoms that did not warrant immediate medical attention.

### Limitations

A total of 70% follow-up was achieved, which is high for a free-standing internet intervention, and there was little evidence of attrition bias. There was no differential attrition bias which suggested that resentful demoralisation was minimised by offering the delayed intervention^21^ ^22^ The primary outcome was initially anticipated to be at 12 months, but a shorter time period was necessary due to the need to engage participants and achieve good follow-up rates with the monthly questionnaires. Monthly questionnaires were also needed, since the intervention during development had to take account of the context of the provision of NHS Direct, and the monthly self-report data was the only source of data about NHS Direct contacts (in addition to documenting episodes that clinicians did not include in the records). The monthly data has the limitations of self-report, and could be biased if GP consultations were discouraged by the Internet Dr, but in fact, the Internet Dr did not gave advice about when to see the doctor promptly. If self-reports of RTIs were biased, we would also have expected different numbers of RTIs to be reported in the intervention group which did not occur. Bias in self-report would also not explain the opposite directions of consultation with NHS Direct and with GPs, and which also makes type I errors unlikely. The estimate derived from primary care notes review for consultations (risk ratio 0.87, lower bound of the 95% CI 0.51) was also consistent with the estimate from the monthly data (risk ratio 0.71). The rate of uptake following invitation was low, but is what would be expected for a free-standing internet-delivered intervention, particularly as this is mostly for minor and common conditions that most will feel, rightly or wrongly, reasonably confident to manage. However, the patients who did participate were those that the intervention is likely to help, that is, participants who are sufficiently concerned about their symptoms to be motivated to use a self-management website. There is also a circularity in engaging participants—physicians are much more likely to refer to a free-standing intervention once it has been shown to be effective, so the first priority is to demonstrate effectiveness. Participants were less deprived than non-participants, but controlling for deprivation made little difference to the estimates, and there was no significant interaction of the intervention with deprivation. The number of participants who experienced one or more RTIs was lower than expected, which will have reduced the power to detect differences. Patients' self-reported contacts with the NHS, but recall of contacts made during an infection experienced in the previous month are likely to suffer minimal recall bias. Self-report is the only method of capturing contacts with NHS Direct and, furthermore, the estimates of consultations and admissions purely based on primary care notes suggested changes in the same direction and of a magnitude similar to the monthly self-reports.

### Main findings

The estimated reduction in consultations with GPs with the website was similar to the effectiveness of the pamphlet we developed for predominantly respiratory illness.^6^ This suggests the internet-delivered intervention is potentially more effective than a pamphlet, given the current widespread availability of NHS Direct online resources and other internet-delivered advice regarding infections. The estimated 25% reduction in GP consultations, even if only over a period of a few months, would provide very considerable relief in terms of pressure on services during the winter months. Perhaps more surprising is that there was a reduction in hospital admissions, albeit non-significant, suggesting the intervention is unlikely to results in delayed presentation of serious illness—and if anything could help in relieving pressure on hospital services. One explanation for reduced admission might be that those with severe symptoms were discouraged from seeing the doctor, but since Internet Dr encouraged individuals to seek medical help promptly with severe symptoms this seems unlikely. Although the study was not powered to assess a reduction in antibiotic use, nevertheless the estimates of a 6–12% reduction in antibiotic prescriptions over 6–12 months is consistent with the observation that most individuals who attend the GP get antibiotics,^1^ so reducing attendance would be expected to potentially provide an important component in the population-level fight against antibiotic resistance, given the evidence that primary-care prescriptions are a key component in driving antibiotic resistance.^3^

### Harms

In terms of major harms, the upper bound of the CI suggests we can be reasonably sure that no increase in hospital admissions occurred. The most surprising finding was that in the intervention group both symptom duration and the duration of more severe symptoms was increased—the latter significantly. This could be either a chance finding or possibly that we made participants more aware of symptoms. However, another possibility is that by strongly encouraging the use of not only paracetamol but also ibuprofen, the intervention may have significantly increased ibuprofen use, and recent trial evidence suggests that advising the use of ibuprofen is unlikely to help overall symptoms, and is associated with the progression of symptoms (ie, prolonging illness)^8^—presumably due to inhibiting the inflammatory element of an effective immune response. When the analysis in the current study controlled for the use of pages that advocated ibuprofen, the finding of increased symptom duration in the intervention group was markedly attenuated. A possible explanation for this attenuation could be that use of ibuprofen pages is a marker of an individual having more severe or florid symptoms, and hence, the symptoms might last longer (ie, reverse causality: the use of ibuprofen pages was because of severe illness, not causing it). However, this explanation is rather unlikely as more florid upper respiratory symptoms and signs are associated with shorter illness duration,^23^ and reverse causality cannot explain why more severe prolonged symptoms were reported in the intervention group since the number of infections reported were almost identical in both groups. Thus, the most reasonable inference is that advice on the use of ibuprofen was probably harmful, and revised versions of the website should therefore not encourage ibuprofen use. Whatever the reasons for the finding of more severe symptoms, the presence of more severe symptoms would be expected to lead to increased consultations, which makes the reduction in the need for GP contacts more striking—supporting the earlier findings in the development of the intervention, that the website increases enablement and confidence in managing symptoms.^12^ ^15^

### Conclusion

An internet-delivered intervention for managing RTIs helps participants appropriately manage their symptoms and contacts with NHS staff, and may help reduce hospital admissions, but advice to use ibuprofen may be unhelpful.
