# Correction

**DOI:** 10.1136/bmjopen-2015-009769corr1

**Published:** 2017-03-21

Little P, Stuart B, Andreou P, *et al.* Primary care randomised controlled trial of a tailored interactive website for the selfmanagement of respiratory infections (Internet Doctor). *BMJ Open* 2016;6:e009769. doi:10.1136/bmjopen-2015-009769

There is a labeling mistake in the analysis of this paper. Although the mistake alters the estimates very slightly it does not alter the inferences.

The misunderstanding was that the 24 week follow-up (the last data point) was thought to be 20 weeks, and the real 20 week data was then not included. The most complete use of the data is therefore to include both the 20 and 24 weeks data. If the complete 24 weeks of data is used throughout this means the following changes to the tables: In the Abstract, the Results section should read: **“Results** 3044 participants were recruited. 852 in the intervention group and 920 in the control group reported one or more RTIs, among whom there a modest increase in NHS Direct contacts in the intervention group (intervention 44/1734 (2.5%) versus control 24/1842 (1.3%); multivariate Risk Ratio (RR) 2.53 (95% CI 1.10 to 5.82, p=0.029)). Conversely reduced contact with doctors occurred (283/1734 (16.3%) vs 368/1845 (20.0%); risk ratio 0.71, 0.53 to 0.95, p=0.019). Reduction in contacts occurred despite slightly longer illness duration (11.3 days versus 10.9 days respectively; multivariate estimate 0.48 days longer (-0.16 to 1.12, p=0.141) and more days of illness rated moderately bad or worse illness (0.53 days; 0.12 to 0.94, p=0.012). The estimate of slower symptom resolution in the intervention group was attenuated when controlling for whether individuals had used webpages which advocated ibuprofen use (length of illness 0.22 days, −0.51 to 0.95, p=0.551; moderately bad or worse symptoms 0.36 days, −0.08 to 0.80, p=0.105). There was no evidence of increased hospitalisations (risk ratio 0.13; 0.02 to 1.01; p=0.051).”

Table 2 is the characteristics of those who reported at least one illness and that does not change. The main impact is on the numbers in Table 3 (monthly report) and Table 4 (notes reviews at 20 weeks in those who reported an infection). These now use 24 weeks of data. There were no changes to the inferences.

**Table 3:** Monthly reports of health service use and duration of illness (4,8,12,16,20,24 weeks) for participants who reported at least one respiratory infection during the 24 weeks

	24 weeks of reported infections and 24 weeks of reported health service use			
	Control	Intervention	Univariate risk ratio (95% CI; p value)	Multivariate risk ratio* (95% CI; p=value)
Reported episodes of respiratory tract infection	1,853/ 6,776 (27.4%)	1,742/6,264 (27.8%)	1.02 (0.93, 1.01; p=0.641)	1.03 (0.94, 1.13; p=0.525)
Of those who reported a respiratory tract infection				
Saw a doctor about illness (as a proportion of the number of episodes)	368/1845 (20.0%)	283/1734 (16.3%)	0.74 (0.53, 0.93; p=0.025)	0.71 (0.53, 0.95; p=0.019)
Contacted NHS Direct about illness	24/1842 (1.3%)	44/1734 (2.5%)	2.39 (1.11, 5.16; p=0.025)	2.53 (1.10, 5.82; p=0.029)
Length of illness (days)	10.88 (9.67)	11.34 (9.91)	0.43 (-0.27, 1.14; p=0.226)	0.48 (-0.16, 1.12; p=0.141)
Days moderately bad or worse	4.16 (5.69)	4.66 (6.89)	0.51 (0.10, 0.92; p=0.015)	0.53 (0.12, 0.94; p=0.012)

*Multivariate model controls for gender, age, highest educational qualification, smoking status, whether there are children aged under 16 years living in the household, any comorbid condition, index of multiple deprivation score, and the number of times the patient reported consulting a doctor about an RTI in the 12 months prior to the study.

**Table 4:** Health service use recorded in primary care records in the 24 weeks following the date of consent for participants who reported at least one episode of respiratory tract infection (RTI)

	24 weeks of reported infections and 24 weeks of notes review data			
	Control (%)	Intervention (%)	Univariate risk ratio (95% CI; p=value)	Multivariate risk ratio* (95% CI; p=value)
Any consultations	127/912 (13.9%)	111/851 (13.0%)	0.94 (0.74, 1.18; p=0.588)	0.92 (0.70, 1.21; p=0.556)
Any antibiotic prescriptions	83/880 (9.4%)	79/827 (9.6%)	1.01 (0.75, 1.35; p=0.953)	1.02 (0.82, 1.43; p=0.911)
Any hospitalisations	8/823 (0.9%)	1/765 (0.1%)	0.13 (0.02, 1.07; p=0.058)	0.13 (0.02, 1.01; p=0.051)
Any referrals	14/824 (1.7%)	12/771 (1.6%)	0.92 (0.43, 1.96; p=0.830)	0.87 (0.35, 2.16; p=0.799)

*Multivariate model controls for gender, age, highest educational qualification, smoking status, whether there are children aged under 16 years living in the household, any comorbid condition, index of multiple deprivation score, and the number of times the patient reported consulting a doctor about an RTI in the 12 months prior to the study.

The title of table 5 is changed as it is actually based on 24 weeks rather than 20 weeks of data. Table 6 is revised to use 24 rather than 20 weeks. No change to the inferences. Table 7 is all notes reviews at 12 months and remains unchanged. Table 8 is characteristics of those lost to follow up which also remains unchanged. The relationship with ibuprofen remains unchanged. (length of illness 0.22, −0.51 to 0.95, p=0.551; moderately bad or worse symptoms 0.36, −0.08 to 0.80, p=0.105).

**Table 5:** Health service use recorded in primary care records in the 12 months following the date of consent for patients who experience at least one episode of respiratory tract infection (RTI) in the first 24 weeks

	Control	Intervention	Univariate risk ratio (95% CI; p=value)	Multivariate risk ratio (95% CI; p=value)
Any reconsultations	176/912 (19.3%)	164/851 (19.3%)	0.99 (0.82 to 1.16; p=0.989)	0.93 (0.73 to 1.16; p=0.509)
Number of reconsultations†	0.36 (1.01)	0.33 (0.85)	0.91 (0.71, 1.17; p=0.475)	0.94 (0.72, 1.21; p=0.619)
Any antibiotic prescriptions	115/851 (13.5%)	107/794 (13.5%)	0.99 (0.78 to 1.30; p=0.982)	1.00 (0.74 to 1.33; p=0.997)
Any hospitalisations	8/748 (1.1%)	1/689 (0.2%)	0.14 (0.02 to 1.08; p=0.059)	0.13 (0.02 to 1.05; p=0.056)
Any referrals	14/750 (1.9%)	12/699 (1.7%)	0.92 (0.43 to 1.96; p=0.830)	0.87 (0.35 to 2.16; p=0.799)

*Multivariate model controls for gender, age, highest educational qualification, smoking status, whether there are children aged under 16 years living in the household, any comorbid condition, index of multiple deprivation score, and the number of times the patient reported consulting a doctor about an RTI in the 12 months prior to the study.

†Reported as the mean (SD). The median is 0 and the IQR is (0, 0). The range is 0–8.

**Table 6.** Health service use in the 24 weeks following the date of consent based on review of primary care notes

	Control	Intervention	Univariate risk ratio (95% CI; p=value)	Multivariate risk ratio (95% CI; p=value)
Any reconsultations	165/1418 (11.6%)	149/1483 (10.1%)	0.86 (0.70, 1.06; p=0.169)	0.86 (0.67, 1.09; p=0.216)
Number of reconsultations†	0.22 (0.82)	0.18 (0.69)	0.82 (0.62, 1.08; p=0.156)	0.89 (0.67, 1.19; p=0.438)
Any antibiotic prescriptions	108/1378 (7,8%)	101/1448 (7.0%)	0.89 (0.68, 1.15; p=0.380)	0.92 (0.68, 1.24; p=0.592)
Any hospitalisations	8/1301 (0.6%)	3/1388 (0.2%)	0.35 (0.09, 1.33; p=0.125)	0.36 (0.10, 1.37; p=0.134)
Any referrals	12/1302 (0.92%)	11/1375 (0.80%)	0.86 (0.38, 1.94; p=0.721)	1.08 (0.42, 2.79; p=0.870)

*Multivariate model controls for gender, age, highest educational qualification, smoking status, whether there are children aged under 16 years living in the household, any comorbid condition, index of multiple deprivation score and the number of times the patient reported consulting a doctor about an respiratory tract infection in the 12 months prior to the study.

†Reported as the mean (SD). The median is 0 and the IQR is (0, 0). The range is 0–8.

The corrected Tables are shown below:

